# Adapting extracellular matrix proteomics for clinical studies on cardiac remodeling post-myocardial infarction

**DOI:** 10.1186/s12014-016-9120-2

**Published:** 2016-09-15

**Authors:** Merry L. Lindsey, Michael E. Hall, Romain Harmancey, Yonggang Ma

**Affiliations:** 1 Mississippi Center for Heart Research, Department of Physiology and Biophysics, University of Mississippi Medical Center, 2500 North State St., Jackson, MS 39216-4505 USA; 2 Division of Cardiology, Department of Medicine, University of Mississippi Medical Center, Jackson, MS USA; 3 Research Service, G.V. (Sonny) Montgomery Veterans Affairs Medical Center, Jackson, MS USA

**Keywords:** Matridomics, Glycoproteomics, Secretomics, Matrix metalloproteinase, Population scale proteomics, Left ventricle, Matrikine

## Abstract

Following myocardial infarction (MI), the left ventricle (LV) undergoes a series of cardiac wound healing responses that involve stimulation of robust inflammation to clear necrotic myocytes and tissue debris and induction of extracellular matrix (ECM) protein synthesis to generate a scar. Proteomic strategies provide us with a means to index the ECM proteins expressed in the LV, quantify amounts, determine functions, and explore interactions. This review will focus on the efforts taken in the proteomics research field that have expanded our understanding of post-MI LV remodeling, concentrating on the strengths and limitations of different proteomic approaches to glean information that is specific to ECM turnover in the post-MI setting. We will discuss how recent advances in sample preparation and labeling protocols increase our successes at detecting components of the cardiac ECM proteome. We will summarize how proteomic approaches, focusing on the ECM compartment, have progressed over time to current gel-free methods using decellularized fractions or labeling strategies that will be useful for clinical applications. This review will provide an overview of how cardiac ECM proteomics has evolved over the last decade and will provide insight into future directions that will drive forward our understanding of cardiac ECM turnover in the post-MI LV.

## Background

The left ventricle (LV) is a complex mixture of cell types, including cardiomyocytes, endothelial and vascular smooth muscle cells, fibroblasts, and immune cells, as well as extracellular matrix (ECM) that surrounds these cell types [1]. Due to the high metabolic demand placed on the myocardium, cardiac myocytes are high energy consumers with 30 % of their volume occupied by mitochondria [2]. Of the protein constituents in the LV, ECM components are present at lower concentrations than mitochondrial or cytoplasmic proteins and generally have lower solubility than intracellular components. Because of this, whole myocardial proteomics is predisposed to evaluating soluble intracellular proteins.

The cardiac ECM provides mechanical support to the LV, coordinates signal transduction, and regulates cell functions [3]. During myocardial infarction (MI), there is extensive extracellular protein turnover as old ECM is replaced by an infarct scar primarily composed of newly synthesized ECM. LV remodeling relies on a balance between ECM clearance and deposition. While excess degradation can lead to LV aneurysms or rupture, excess deposition can lead to a stiff LV that provides a substrate for the development of heart failure or arrhythmias [4–6]. Evaluating ECM, therefore, is important for our complete understanding of LV remodeling.

Over the last 10 years, proteomic capabilities have dramatically increased due to technological improvements, including enhanced sample preparation protocols and improved capabilities in mass spectrometry (MS), database searching, and bioinformatics analysis of results. Combined, these improvements have made the evaluation of cardiac ECM more approachable. In this review, we will summarize the current state of the field and provide examples of how ECM proteomics is being used to better understand post-MI remodeling and to evaluate plasma and tissue samples from human subjects following MI. We will also borrow from the sepsis field to illustrate recent advances in glycoproteomics that have applicability for MI studies.

## Indexing ECM proteins expressed in the LV

The first hurdle encountered was the fact that myocardial samples have a predominance of intracellular constituents, which made it very difficult to even identify ECM proteins, let alone quantify them. In the setting of MI, using samples that include the entire LV (both remote and infarct regions) focuses the evaluation on easily soluble mitochondrial and cytoplasmic proteins. While the analysis of a whole tissue sample is the simplest method, this approach is not designed to focus on ECM.

To overcome this issue, we used another approach that takes advantage of the natural overabundance of collagen in the newly formed scar to target and enrich samples in ECM proteins. By day 7 post-MI, the infarct region is composed of 30 % collagen and can be easily visualized by picrosirius red staining [7, 8]. We used this biochemical property to our advantage and excluded the non-infarcted remote region during sample collection. By selecting only the infarct region and evaluating its protein composition by two dimensional gel electrophoresis (2DE gels), we were able to identify multiple ECM proteins, including fibronectin, laminin, peroxiredoxin-1, -2, and -3, tenascin-C, and thrombospondin-1 [9, 10]. Comparing wild type mice to mice with global genetic deletions for matrix metalloproteinase (MMP) genes, we were able to identify several MMP-7 and MMP-9 substrates based on differences in expected and actual molecular weights [9–11]. Of these, fibronectin was demonstrated to be an in vivo MMP-7 and MMP-9 substrate in the infarcted LV. While this approach was an improvement over previous attempts using whole myocardial samples, the presence of a large amount of intracellular proteins still made the detection of lower abundance ECM proteins difficult.

To further remove the intracellular constituents and enrich for ECM, we and others have used decellularization approaches, primarily involving the incubation of samples in 1 % sodium dodecyl sulfate (SDS) [12]. The Mayr laboratory developed a sequential extraction methodology for cardiac ECM, which involves tissue decellularization using SDS followed by re-solubilization and centrifugation to fractionate protein according to solubility [13]. These investigators used this approach in a pig model of ischemia/reperfusion to identify for the first time novel ECM proteins that contribute to cardiac remodeling [14]. This list included cartilage intermediate layer protein 1, matrilin-4, extracellular adipocyte enhancer binding protein 1, collagen alpha-1(XIV), and several members of the small leucine-rich proteoglycan family, including asporin and prolargin. Further analysis of over 100 ECM proteins revealed signatures of early- and late-stage cardiac remodeling, with transforming growth factor-beta1 signaling at the epicenter of the interaction network. This biosignature of early- and late-stage ECM remodeling after myocardial ischemia/reperfusion injury has clinical utility both as prognostic markers and modifiable targets for drug discovery. Identifying different biosignatures in ECM remodeling post-MI could be used to determine whether a patient will recover and to what extent. Identifying patients with biosignatures that indicate insufficient or overly robust wound healing could be useful in determining which patients are at risk and need to be followed-up more closely and treated more aggressively.

Our team has also used this approach, which we termed the Texas 3 step protocol, to enrich for low-abundant and insoluble ECM proteins [12]. After decellularization, we used acid extraction and enzymatic deglycosylation to enhance re-solubilization. The end output was the generation of three fractions consisting of proteins of decreasing solubility. The fact that these fractions were obtained using buffers that are MS-compatible allowed downstream proteomic analysis. One issue with using this approach for cardiac samples is that, due to the many fractionation steps with protein loss along the way, it is difficult to obtain reproducible quantification. While protein loss during extraction could be corrected by spiking the sample with a known standard, this approach is better used for identification of the different types of ECM proteins present in a sample. Decellularization is more difficult to achieve in cardiac tissue compared to vascular tissue, where it has also been used to successfully identify ECM components and quantify major changes such as presence versus absence differences and substantial fold changes [13, 15]. Comparing across fractions provides details on ECM release, solubility, and interaction strength.

## Quantifying amounts

Quantitation can be achieved using both label and label-free approaches [16, 17]. Of particular relevance to ECM studies, *N*-linked glycopeptides isolated from tryptic peptides using a solid phase extraction of glycopeptides (SPEG) method has been used to identify and quantify novel ECM changes in the post-MI setting [18, 19]. Our team has recently employed the gel-free SPEG approach using lectin affinity chromatography to isolate out and quantify glycosylated proteins in both plasma and tissue samples [20–22]. Peptides were quantified by label-free relative quantification based on integrated peptide peak intensities. This approach has the strength that it eliminates highly abundant, non-glycosylated proteins such as albumin. Enriching for glycoproteins is also advantageous when evaluating ECM, as ECM proteins as a whole are highly glycosylated [20]. Of note, MMP catalytic sites contain a conserved *N*-linked glycosylation site and do not contain O-linked glycans [23]. In the post-MI LV, glycoproteomics provides a powerful platform on which to identify and quantify *N*-glycosylated proteins important for LV remodeling [22]. Out of 541 *N*-glycosylated proteins quantified, we showed that CD36 is a novel substrate cleaved by MMP-9, with important implications for macrophage phagocytosis in the MI setting. A limitation of this approach is that the differences in amounts refers to the amount of glycosylated protein and may not reflect total protein amounts. A change in glycosylation would register as a difference, while total protein concentration may be unchanged. As with any discovery proteomics workflow, SPEG requires validation by an independent technique such as multiple reaction monitoring, ELISA, or immunoblotting [24, 25].

In isolated cell culture, ECM secretion assessed by MS is termed secretomics. This gel-free approach has been used to evaluate miRNA effects on ECM secretion by cardiac fibroblasts [26]. In this study, the secretome from mouse cardiac fibroblasts transfected with the pre-/anti-miR for miR-29b was analyzed by MS and quantified as spectral counts. The miR-29b targeted fibrosis-related proteins, including collagens, MMPs, leukemia inhibitory factor, insulin-like growth factor 1, and pentraxin 3 (PTX3). In the pre-miR-29b overexpression group, for example, fibulins 3 and 4 and TIMP-2 were increased while collagen IA1 and IA2 and MMP-2 were decreased. After transfection with pre-miR-29b, the conditioned medium of cardiac fibroblasts lost the ability to support cardiac myocyte adhesion ex vivo. This proteomic analysis revealed novel molecular targets of miRNAs linked to fibrosis. Such comprehensive screening methods are critical components for assessing cardiac ECM [26]. The Mayr laboratory has also used secretomics coupled to glycoproteomics to analyze the secretome of human endothelial cells, using a similar gel-free approach and quantifying using spectral counts [27]. This study provides the most comprehensive catalogue of endothelial protein secretion to date and demonstrates the potential of using a workflow that combines higher-energy C-trap dissociation with electron-transfer dissociation for a hybrid linear ion trap-orbitrap mass spectrometer that can determine the glycosylation status of complex biological samples.

## Qualitative characterization

One issue with gel-free approaches is that molecular weight and pH information is not provided, which makes it more difficult to infer post-translational modifications using unbiased approaches. Post-translational modifications relevant to ECM include cross-linking, glycosylation, and proteolytic processing by MMPs. MS techniques are currently robust enough to provide information on protein–protein interactions that occur over the time course of MI. Using global approaches for large-scale profiling can allow one to catalogue protein identities and relative concentrations. Identifying potential protein partners can then be followed up with other methodologies to provide validation. In order for the protein–protein interaction to be mapped using MS technology, the intact protein complex needs to be first purified by affinity purification before digestion and MS analysis [28].

Proteomics can also be used to provide information about membrane topography [20]. The ECM proteome consists of all proteins expressed outside the cell, including cell surface and secreted proteins; and due to their location, ECM proteins are highly amenable drug targets [29]. Information on protein location and availability can provide insight useful for selecting optimal antigen sites and identifying drug targets. One common feature that is highly desirable is easy accessibility, making ECM protein analysis highly relevant to drug development [30]. For this reason, efforts to determine the spatial orientation of target proteins have been undertaken. In addition to traditional methods for topology evaluation, membrane protein site accessibility is becoming a useful tool to evaluate protein orientation. These methods include evaluation of *N*-glycosylation sites, antibody epitopes, iodinatable sites, and proteolytic sites [30]. Of these, *N*-glycosylation site assessment can be used for topology evaluation because *N*-linked glycosylation occurs only in extracellular domains of membrane proteins [31].

*N*-linked glycosylation site analysis can provide in vivo topologic evidence that has not been made available by other approaches. Zielinska and colleagues showed that glycosylation sites of membrane proteins always orient toward the extracellular space [32]. In fact, Gundry and co-workers used this concept to determine that the transmembrane glycoprotein ZIP14 had been assigned an orientation by Swiss-Prot that was incorrect, and this annotation has since been corrected [31]. We have used a similar approach to identify 1352 unique *N*-linked glycosylation sites and provide new topologic information on hundreds of ECM proteins [20]. In the β_1_ integrin, for example, there were 14 predicted *N*-linked glycosites, with only four having been previously identified (N363, N366, N376, and N669). We identified four novel *N*-glycosites for β_1_ Integrin (N212, N406, N481, and N520) that have recently been confirmed by other groups [33, 34]. These sites are distributed throughout the predicted extracellular domain, which validates this section being extracellular and helps to eliminate false positives.

## Adapting these approaches to clinical samples

Using proteomics to analyze clinical samples shares many of the same technical considerations as pre-clinical studies. In assessing feasibility, there are factors that should be taken into account, including the scope of the intended study (broad or targeted) for which different technologies may be used. This, in turn, will also influence downstream factors, such as the number of groups to compare and the number of replicates that can be processed in a manageable experiment. These considerations are listed in Table 1 and include criteria for subject selection, sample type, collection method, sample storage conditions, data acquisition and analysis methods, and results documentation.

**Table 1:** Experimental design considerations for clinical studies. Modified from [39]

Criteria for subject selection
Type of samples and method of collection
Storage conditions of the sample
Methods of data acquisition and analysis
Documentation of results
Reproducibility and replication assessment

### Reproducibility and replication assessment

The first step involves assessing project feasibility and developing a careful experimental design. In terms of project feasibility, a major consideration is how to harness the experiment to keep acquisition, analysis, and data interpretation feasible. If there are a large number of expected output measurements, limiting the number of inputs (e.g. group number) will help to maintain feasibility and provide adequate power for statistical analysis. Experimental designs that compare more than four groups often suffer from power that is reduced to below acceptable limits and make the experiment unwieldy. For example, it would be better to have 2 groups with n = 10 per group than 4 groups with n = 5 per group. Increasing the biological replicates for the MS analysis can be coupled to downstream validation experiments, such as enzyme-linked immunosorbent assay or immunoblotting, which can more easily accommodate additional controls. If using a targeted proteomics approach with a limited number of expected outputs, then the number of inputs can be increased to include a broad number of controls. Targeted proteomics refers to the analysis of a preselected group of proteins [35]. As techniques evolve, the use of population scale proteomics will be possible for the more general proteomics lab.

### Sample collection and storage

Other considerations for experimental design are sample collection and storage conditions and potential solubility issues. With many clinical cohorts, samples were collected before we understood the importance of collecting samples in protease inhibitors. There have been considerable efforts made to develop uniform collection standards for plasma proteomics experiments to improve both reproducibility and replication. As part of the Human Plasma Proteome Project, evaluation of serum and plasma samples collected under a variety of conditions showed that EDTA anti-coagulated plasma samples gave the most reproducible results [36]. Of note, however, the results of this study were highly variable, in part due to a lack of laboratory selection criteria and analytical standards to assess results quality. Another issue with this approach is that by developing a global approach, individual priorities may not be met. Downstream applications often require the use of a specific reagent. For example, MMPs are best studied in plasma samples collected with heparin as the anti-coagulant, because EDTA inhibits MMP activity [37, 38]. Therefore, plasma proteomics for ECM may not be entirely compatible with a general consensus protocol. If the amount of sample is not a limiting factor, we recommend using multiple recovery techniques in a protocol (e.g. obtain two blood samples from the same individual: one in a heparinized tube and another in an EDTA-treated tube) to maximize the amount of information that one can obtain. In addition, because the plasma potentially reflects changes across all organs, determining cardiac source or cardiac relevance is important.

In terms of storage, samples should be matched across groups in regards to storage conditions, including length and temperature. If storage has been prolonged, evaluating the sample condition (e.g., by running 10 μg total protein on a 1DE gel to assess for degradation) is crucial. For tissue samples, many ECM proteins are highly insoluble, and buffer selection for protein extraction is important and depends on the question being addressed. Consideration should be given for whether samples are immediately analyzed after collection or stored and examined as batch analysis. While analyzing immediately would remove variation due to storage times, whether analysis can be grouped across batch runs would need to be assessed by the individual proteomics facility and would need to include robust statistical support. In our studies, we have not compared results across batches, but rather have collected samples in protease inhibitor cocktail to minimize degradation during storage.

If enrichment is needed to quantify lower abundance proteins, the buffer used may differ from that used for experiments where the goal is to provide an inventory of all proteins present in the sample. This is not a concern for plasma samples, since the proteins are already in a soluble media.

Once the experimental design is developed, downstream acquisition, analysis, and validation are the final steps. Considerations for these components of the experiment have been well described elsewhere [39–41]. Below, we provide examples of how cardiac ECM proteomics has been used in clinical studies. We and others have used proteomics in translational research to catalogue proteins present during pathology, as well as to determine protein functions and explore protein–protein interactions.

### Applications

**Fig. 1:**
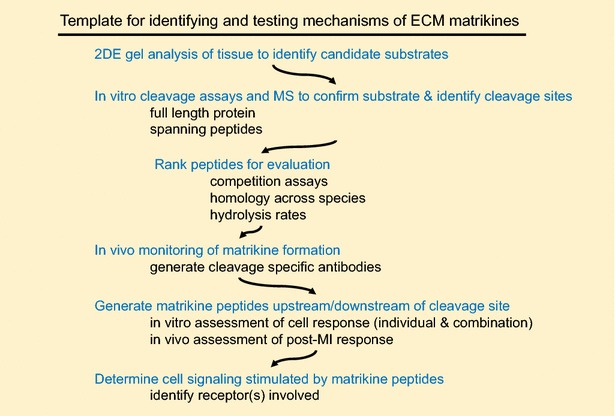
An example of one project work flow for extracellular matrix proteomics experiments that start with 2DE-gel evaluation and mass spectrometry experiments and culminate with functional assays to provide mechanistic insight

We have used the natural overabundance of ECM protocol to identify a novel MMP-9 cleavage site on collagen I (at amino acids 1158/1159) in the LV infarcts of mice [11]. We took this observation and showed that a fragment was generated by MMP-2 and MMP-9, with MMP-9 further degrading it. ECM fragments that are generated by proteolysis or other processing and have biological activity in addition to or distinct from the parent ECM protein are termed matrikines or matricryptins [42]. To test whether the collagen matrikine had signaling activity, a peptide 20 amino acids downstream of the cleavage site was generated and tested for in vitro and in vivo activity. Fibroblasts stimulated with this peptide showed enhanced wound closure properties. Human umbilical vein endothelial cells stimulated with the p1158/59 peptide, but not the spanning peptide, has increased tube formation. Injection of the p1158/59 peptide at 3 h post-MI attenuated LV dilation by promoting angiogenesis and limiting myofibroblast activity. Injection of this peptide at 3 h post-MI attenuated LV dilation by promoting angiogenesis and promoting myofibroblast wound healing activity. In humans with MI, higher concentrations of this collagen fragment associated with improved LV function. This set of experiments provides a template on which to design proteomics studies to evaluate additional ECM matrikines generated by MMPs post-MI (Fig. 1).

Decellularization has been used to evaluate the ECM composition of human samples, including samples from human abdominal aneurysms and other vascular pathologies [14, 15, 43–46]. The Mayr laboratory has used this approach coupled with sequential extraction to identify hundreds of ECM proteins in human aortas, human abdominal aortic aneurysms, and human LV from patients with ischemic cardiomyopathy [13–15, 45, 47]. This team has been the first to report the presence of more than a dozen ECM proteins not previously identified in the myocardium. By combining protein signature evaluation during both acute and chronic remodeling time points with protein network interaction analysis, Mayr and colleagues identified transforming growth factor β1 as being a pivotal regulator of ECM remodeling induced by ischemia and reperfusion [14]. Decellularization approaches provide an unbiased first pass method to focus on ECM changes that are cause and consequence of LV remodeling.

Our team used glycoproteomics to show that sepsis outcomes are linked to the activation of distinct proteins present in the blood coagulation pathways [21]. In our cohort of 20 patients, we evaluated plasma collected at the time of admission and subdivided groups based on survival outcomes. We identified 234 glycoproteins; of which 54 were unique to the survivor group, 43 were unique to the non-survivor group, and 137 were common responses between groups. By immunoblotting, plasma samples from non-survivors showed elevated total kininogens and reduced total cathepsin-L1, vascular cell adhesion molecule, periostin, neutrophil gelatinase-associated lipocalin, and glycosylated clusterin compared to the survivor group. Kyoto Encyclopedia of Genes and Genomes analysis revealed that survivors relied on the extrinsic pathway of the complement and coagulation cascade, while non-survivors relied on the intrinsic pathway.

Cuello and colleagues used an endotoxemic mouse model coupled to a tissue-based proteomics approach for biomarker discovery and identified PTX3 as a lead candidate for inflamed myocardium during sepsis [48]. They found that PTX3 accumulation as an octamer was due to disulfide-bond formation, which was present in heart, kidney, and lung. Of interest, PTX3 oligomeric moieties were also detectable in the circulation. They expanded on these results in their animal model to demonstrate that from day 2 after admission, octameric to monomeric PTX3 conversion consistently associated with a greater survival after 28 days of follow-up. Further, monomeric PTX3 was inversely associated with the extent of cardiac damage, indicated by elevated NT-proBNP and high-sensitivity troponins I and T. Relative to the conventional measurements of total PTX3 or NT-proBNP, the oligomerization of PTX3 showed superior performance in predicting disease outcomes. This provides an excellent example of how to start with a proteomics observational experiment and carry it forward to provide mechanistic links.

The Overall laboratory has successfully used terminomics analysis of protease cleavage sites as a degradomics substrate discovery platform [49]. This approach uses terminal amine isotopic labeling of substrates (TAILS) as a high-throughput method to identify protease-generated neo-N termini. Using negative selection, the TAILS approach first enriches for all *N*-terminal peptides and then uses primary amine labeling-based quantification to discriminate. *N*-terminal and lysine amines are blocked by dimethylation (formaldehyde/sodium cyanoborohydride) and labeled for relative and absolute quantification. After tryptic digestion, a high molecular weight dendritic polyglycerol aldehyde polymer binds the internal tryptic and C-terminal peptides, which now have *N*-terminal alpha amines. This provides the means for *N*-terminal peptide separation. The unbound naturally blocked peptides are recovered by ultrafiltration and analyzed by tandem mass spectrometry (MS/MS). This labeling approach is versatile and well-suited to a broad range of applications, including in vitro cell culture analyses and in vivo tissue analyses. Hierarchical substrate winnowing can be used to subtract out background proteolysis products and non-cleaved proteins.

In translational studies, proteomics often serves as the beginning experiment from which to base a mechanistic evaluation of the system. With clinical samples, proteomics often serves as the final experiment from which to evaluate outputs in a trial or to identify novel biomarkers that can then be taken back into basic science projects in reverse translational study applications.

## Conclusions and future directions

Table 2 summarizes the evolution of approaches we and others have taken over the years to evaluate the cardiac ECM proteome and provides strengths and limitations for each approach. The best approach will depend on the question being asked, and experimental designs can vary depending on the importance placed on more complete cataloguing, quantification, or assessing targeted protein quality. Often, a combination of methods is used through a study to provide a more comprehensive assessment. For example, 2DE gel analysis for first pass identification is often a great starting point, with targeted proteomic strategies used as follow-up experiments to provide mechanistic insight. Of note, the need for bioinformatics is important, in order to process the massive amount of data generated from proteomic approaches.

**Table 2:** Evolution of approaches used to examine cardiac extracellular matrix [9, 10, 12, 20, 22]

Approach	Strengths	Limitations
1. Whole LV proteomics	Routine protocol; fewest processing steps minimizes loss	Selects for highly abundant proteins, which are mostly intracellular
2. Infarct region only proteomics	Uses natural accumulation of ECM that occurs during scar formation	Intracellular proteins still in high abundance
3. Decellularization proteomics	Allows identification of a wide variety of proteins	Difficult to reproducibly quantify
4. Glycoproteomics	Provides reproducible quantification of glycosylated proteins	Selects for glycosylated proteins Molecular weight and pH information not provided

In conclusion, methods to catalogue cardiac ECM using proteomics technologies have dramatically improved over the last 10 years. Current research in the post-MI cardiac ECM field is focused on defining the mechanistic roles for these identified proteins. Of particular importance are the signaling cascades initiated by ECM matrikines generated by MMP proteolysis. In the clinical field, translation of both these approaches and findings will help to identify novel targets and mechanisms of post-MI remodeling.
